# Evaluation of Bladder Dysfunction Outcomes Among Standardized Bladder Shapes in Children With Spina Bifida

**DOI:** 10.1002/nau.70131

**Published:** 2025-08-25

**Authors:** Zoe S. Gan, Joey Logan, Ariana L. Smith, David Ostrowski, Christopher Long, Dana Weiss, Jason Van Batavia, Stephen Zderic, John Weaver, Gregory Tasian

**Affiliations:** ^1^ Division of Urology, Department of Surgery University of Pennsylvania Perelman School of Medicine Philadelphia Pennsylvania USA; ^2^ Department of Biomedical and Health Informatics The Children's Hospital of Philadelphia Philadelphia Pennsylvania USA; ^3^ Department of Surgery, Division of Urology The Children's Hospital of Philadelphia Philadelphia Pennsylvania USA; ^4^ Department of Urology Cleveland Clinic Children's/Cleveland Clinic Lerner College of Medicine Cleveland Ohio USA; ^5^ Department of Biostatistics, Epidemiology, and Informatics Perelman School of Medicine at the University of Pennsylvania Philadelphia Pennsylvania USA

**Keywords:** neurogenic bladder, neurogenic, pediatrics, spinal dysraphism, urodynamics, urology

## Abstract

**Objectives:**

To (1) propose standardized terminology for bladder shapes on fluoroscopic images in a pediatric spina bifida population and (2) determine if bladder shape is associated with filling pressures and other measures of bladder dysfunction. We hypothesized that oblong, trabeculated, and “Christmas tree” bladders would have higher filling pressures and worse bladder function (higher filling pressures; higher presence of vesicoureteral reflux, leakage, detrusor‐external sphincter dyssynergia, and hydronephrosis) than smooth and round‐shaped bladders.

**Methods:**

We conducted a cross‐sectional study of pediatric and adolescent patients with spina bifida who underwent video urodynamics (VUDS) at a tertiary center from July 2016 to June 2022. Representative fluoroscopic bladder images from the earliest available VUDS were categorized by unsupervised cluster analysis. Five urologists also determined standardized classifications for bladder shape (round, oblong, and “Christmas tree”) and contour (smooth or trabeculated/diverticulated), which were applied to the bladder images. Bladder filling pressures and clinical measures of bladder function were compared among bladder shapes.

**Results:**

Four hundred seventeen patients with a median age of 2.6 years (IQR 0.4–8.0 years) were included. For the machine learning cluster analysis of bladder shape, clusters with more trabeculated‐appearing bladders had higher filling pressures. For expert clinician classification, round trabeculated, oblong trabeculated, and “Christmas tree” bladders had higher filling pressures than round smooth and oblong smooth bladders, a difference that was statistically significant. Statistically significant differences were noted among bladder shapes for the presence of vesicoureteral reflux, leakage, detrusor‐external sphincter dyssynergia, and hydronephrosis. Moderate and severe bladder dysfunction were present across all bladder shape clusters.

**Conclusions:**

We established a standardized bladder shape nomenclature in children and adolescents with spina bifida. Higher bladder filling pressures are associated with trabeculations and “Christmas tree” appearance versus smooth contour, but not oblong versus round shape. Bladder shape alone does not appear to consistently differentiate the presence of hydronephrosis, vesicoureteral reflux, leakage, detrusor‐external sphincter dyssynergia, or bladder dysfunction severity.

## 1 Introduction

Fluoroscopic bladder imaging is performed during video urodynamics (VUDS) and voiding cystourethrogram (VCUG) as part of bladder function evaluation. These images provide an assessment of bladder shape, bladder neck appearance, and the presence or absence of vesicoureteral reflux (VUR). However, the overall utility of fluoroscopy in the evaluation and management of bladder dysfunction is not well defined. An International Consultation on Incontinence Research Society Think Tank concluded in 2014 that the indication for bladder imaging during VUDS is unclear, and that the risk‐benefit balance of fluoroscopic exposure needs to be better defined and evaluated [1]. Accordingly, there is a need to better understand the importance of bladder shape alongside urodynamic data.

Understanding the role of bladder shape requires standardization of shape classification and terminology, which has not been established. Existing approaches in the literature include overall bladder deformity grading without adequate instructions on how to classify the degree of deformity [2], trabeculation grading based on the amount of bladder surface involved without accounting for other shape characteristics [3], and limited binary classifications of bladder shape as “smooth and round” versus not [4] or “atypical” versus “typical” [5]. Standardizing bladder shape nomenclature would facilitate defining the potential diagnostic and prognostic values of bladder shape. Such information would be particularly beneficial for pediatric patients with spina bifida, as these patients often have early bladder dysfunction and undergo numerous VUDS studies over the course of their lifetime.

Artificial intelligence, particularly machine learning (in which a computer learns from data to perform tasks), has become increasingly utilized in urology. Clinical applications of machine learning include optimizing patient workflow, enhancing analysis of pathological and radiological images, and improving diagnostic accuracy and outcome prediction [6, 7]. While machine learning is limited by the quality of training data, potential overfitting, and lack of transparency, it can integrate large amounts of data to identify hidden patterns. This ability may be helpful for identifying certain bladder shape phenotypes.

The purposes of this study were 1) to propose standardized terminology for bladder shapes in a pediatric spina bifida population, and 2) to determine if bladder shape, as classified by both unsupervised cluster analysis of fluoroscopic images and expert clinicians, is associated with filling pressures and measures of bladder dysfunction. We hypothesized that oblong, trabeculated, and “Christmas tree” bladders would have higher filling pressures and more bladder dysfunction than smooth and round bladders. The correlation of standardized bladder shape with clinical outcomes of interest may ultimately allow for improved assessment of bladder dysfunction severity and prognosis of both bladder and renal function in pediatric and adult populations.

## 2 Materials and Methods

### 2.1 Study Design and Population

This cross‐sectional study was approved by the Institutional Review Board at The Children's Hospital of Philadelphia. We included patients under age 18 years with spina bifida who were evaluated and whose data were prospectively collected under a clinical protocol at The Children's Hospital of Philadelphia between July 2016 and June 2022. We extracted data from their earliest available VUDS study and the electronic medical record. Demographic and clinical data included age at the time of VUDS, sex assigned at birth, race, ethnicity, weight, estimated bladder capacity (EBC; calculated as weight in kilograms x 7), current use of clean intermittent catheterization (CIC), and current use of anticholinergics. We excluded patients who had fluoroscopic images in which no bladder was identified, did not reach 25% of weight‐based EBC during filling, had prior bladder augmentation, or had missing outcome data. Filling was performed via pump at 4% EBC per minute.

### 2.2 Bladder Shape Classification

All patients had a VCUG done during VUDS. For each VUDS study, fluoroscopic images were taken in the anterior‐posterior view. A computer program was used to identify the bladder defined by contrast in each fluoroscopic image and select the image with the median bladder area as a representative image for bladder shape about halfway through the filling process. These representative images were manually reviewed to confirm adequate capture of bladder shape. Any image in which the bladder shape was not adequately captured (e.g., blurry, bladder out of frame, etc.) was exchanged for another image within the same VUDS study that best captured bladder shape as determined visually by clinician review.

Representative fluoroscopic images of bladder shape were classified by two independent methods: 1) by a computer using a machine learning method of unsupervised cluster analysis, and 2) by urologists using manual review. For computer classification, images were passed through a DenseNet deep learning model pre‐trained on hundreds of thousands of natural images, and the global average pooling layer was extracted as a single array. A K‐means clustering algorithm was applied to the arrays to group similar images into clusters using a pre‐determined number of five clusters for clinical utility. All models were created using Python Version 3.9.13.

Before bladder shape classification by manual review, five expert reviewers (four pediatric urologists and one specialist in urogynecology and reconstructive pelvic surgery) first collaboratively determined standardized classifications for bladder shape (round, oblong, and “Christmas tree”) and contour (smooth or trabeculated/diverticulated) based on clinical expertise and existing literature. It was determined that qualifying for “Christmas tree” shape also required a contour that was trabeculated and/or had diverticuli. Oblong shape was defined as length at least twice the width or vice versa. Both oblong and round shapes could have either smooth or trabeculated/diverticulated contour. Bladder contour was determined based on the majority of the contour (e.g., if 70% of the bladder shape contour appeared trabeculated and 30% appeared smooth, then the contour was designated as trabeculated).

Next, two authors (ZG and DO) were oriented to the standardized classification system and independently designated the shape and contour of each bladder image. Cohen's kappa coefficient was used to measure inter‐rater reliability for bladder shape, contour, and overall category based on both shape and contour. The strength of agreement was deemed slight for values 0–0.20, fair for values 0.21–0.40, moderate for values 0.41–0.6, substantial for values 0.61–0.80, and almost perfect for values 0.81–1.00 [8]. Tiebreak was performed by a third author (JW) for any differences.

### 2.3 Outcomes

The primary outcome was bladder pressure during filling, which was calculated as total intravesical pressure minus abdominal pressure to isolate detrusor pressure. Bladder pressure was measured at 25%, 50%, 75%, and 100% of EBC as the bladder was being filled. Secondary outcomes reflecting bladder dysfunction included the presence of the following during VUDS: VUR, leak, and detrusor‐external sphincter dyssynergia (DESD). Additional secondary outcomes included the presence of baseline hydronephrosis (determined by the renal bladder ultrasound performed closest to the time of VUDS) and the overall degree of bladder dysfunction (none to mild, moderate, or severe). The severity of bladder dysfunction (mild, moderate, or severe) had been previously determined for a subset of 284 VUDS studies by the five expert reviewers based on several factors such as pressure and volume tracings, bladder compliance, detrusor leak point pressure, DESD, detrusor overactivity, VUR, and bladder appearance; these methods are reported in a previous study from our group [9]. The degree of bladder dysfunction was designated based on the principle that worsening bladder dysfunction increases the risk of renal dysfunction, and that this risk drives management decisions such as starting CIC or offering bladder augmentation. Finally, the filling pressure and proportion of EBC at which leakage occurred during VUDS were reported for each bladder shape.

### 2.4 Statistical Analysis

For each category of bladder shape, the primary outcome of bladder filling pressure was summarized at 25%, 50%, 75%, and 100% of EBC using mean and standard deviation. Bladder pressures at each filling level were compared pairwise between bladder shapes using Wilcoxon‐Mann‐Whitney tests. The majority of secondary outcomes (VUR, leak, DESD, and hydronephrosis) were summarized using proportions and compared pairwise between bladder shapes using Chi‐square tests. The remaining secondary outcome (overall degree of bladder dysfunction) was compared descriptively (no statistical comparisons) between bladder shape clusters. All pairwise tests incorporated Bonferroni corrections for multiple comparisons. *p* values less than the Bonferroni‐corrected threshold of 0.005 were considered significant.

Several sensitivity analyses were performed. First, pairwise analyses were repeated excluding patients who were on anticholinergic medication or CIC, as well as patients with a history of bladder Botox or ureteral reimplantation. This was performed to determine if factors potentially affecting bladder shape would influence results. Second, among bladders with each degree of bladder dysfunction (none to mild, moderate, or severe), selected secondary outcomes (VUR, leak, DESD, and hydronephrosis) were compared between pairs of bladder shape clusters using pairwise chi‐square tests with Bonferroni corrections. This was performed to determine if bladder shape could differentiate these outcomes for a given level of bladder dysfunction. Third, silhouette analysis, in which the quality of each cluster is measured based on maximizing both similarity within the cluster and difference from neighboring clusters, was used to determine the optimal number of clusters (K) [10]. The cluster analysis was repeated using this optimal number of clusters, and the selected secondary outcomes were compared between clusters.

## 3 Results

### 3.1 Population Characteristics

We identified 487 patients with spina bifida who underwent VUDS at our institution from July 2016 to June 2022. We excluded 30 adults, two patients with prior history of bladder augmentation, 27 patients who had incomplete data (either did not reach 25% of weight‐based EBC during filling or had missing outcome data), and 11 patients who had fluoroscopic images in which no bladder was identified. We ultimately analyzed the first available VUDS study from 417 patients. Demographic and clinical characteristics for the cohort are summarized in Table 1. All patients had previously had their spina bifida closed by a neurosurgeon.

**Table 1:** Demographic and clinical characteristics of the study cohort at the time of the first available videourodynamics study. Unless otherwise specified, summary measure refers to median (interquartile range) for continuous variables and *n* (%) for categorical variables.

Characteristic	Summary measure
Age (years)	2.6 (0.4–8.0)
Age range	1.6 months to 17.2 years
Female sex	218 (52%)
Race	
White	269 (65%)
Black	34 (8%)
Asian	19 (5%)
Multiracial or other	92 (22%)
Unknown	3 (1%)
Ethnicity	
Hispanic or Latino	73 (18%)
Unknown	2 (1%)
Weight (kilograms)	12.0 (6.75–25.0)
Estimated bladder capacity (milliliters)	84.0 (47.25–175.0)
On clean intermittent catheterization	9 (2%)
On anticholinergic medication	121 (29%)
History of bladder Botox	7 (2%)
History of ureteral reimplantation	3 (1%)
Spina bifida type	
Myelomeningocele	338 (81%)
Lipomeningocele	60 (14%)
Myeloschisis	13 (3%)
Unknown	6 (1%)

### 3.2 Bladder Shape Classification

**Figure 1:**
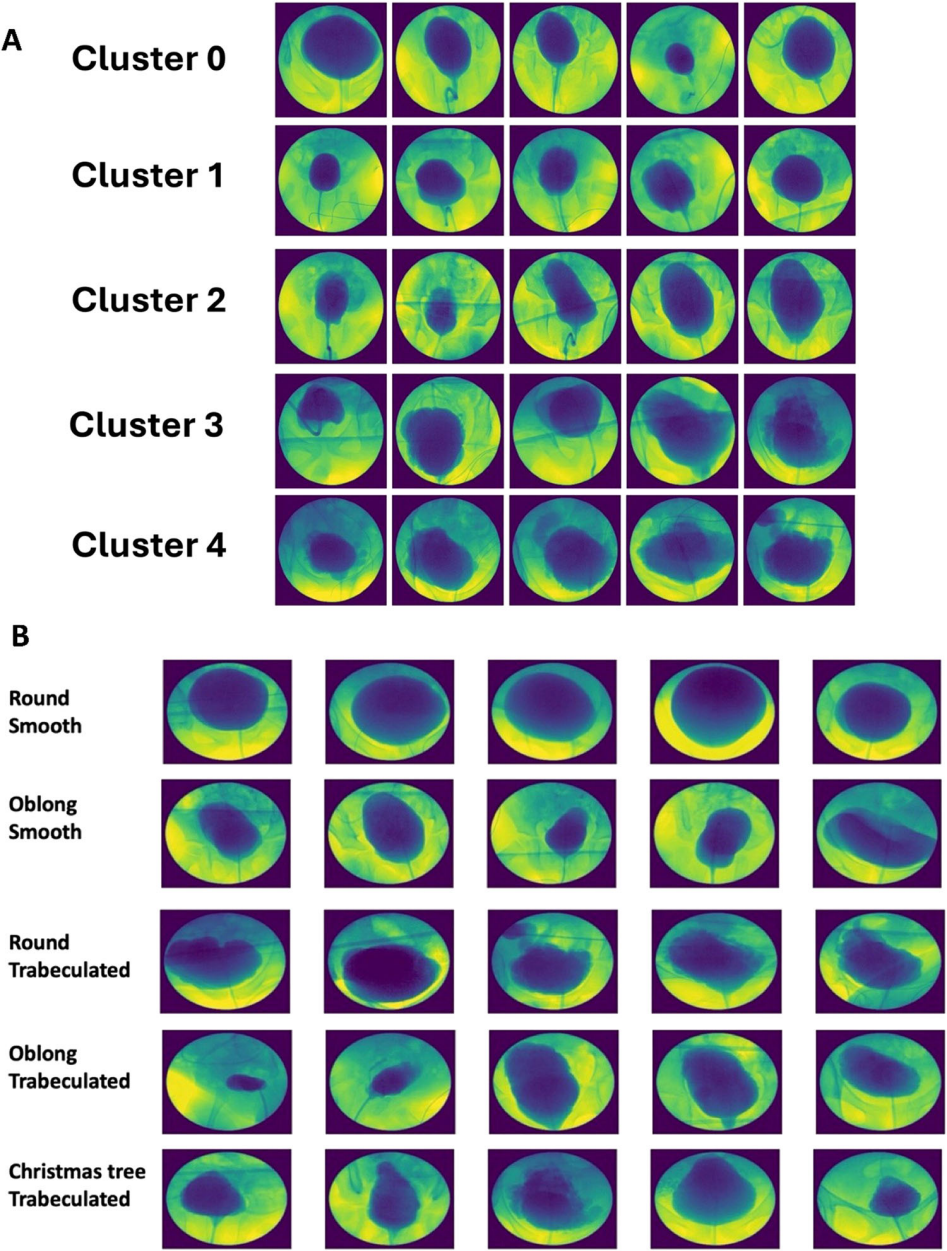
Sample fluoroscopic images from each (A) bladder shape cluster determined by machine learning and (B) category assigned by expert clinicians.

We manually selected the representative fluoroscopic image for 12 studies (2.7%) in which the bladder image that was automatically selected was determined by clinicians to be inadequately captured. Sample fluoroscopic images are shown for both bladder shape clusters determined by the machine learning algorithm (Figure 1A) and clinician‐determined categories (Figure 1B). For the machine‐generated clusters, bladders in Clusters 0 and 1 generally appeared smooth and round, bladders in Cluster 2 appeared oblong, and trabeculations were noted more frequently in Clusters 3 and 4. For clinician‐determined categories (Table 2), bladders were most commonly categorized as round smooth (74%), followed by round trabeculated (10%), oblong smooth (9%), “Christmas tree” (4%), and oblong trabeculated (3%). There was substantial agreement between raters for bladder shape (*k* = 0.74) and both bladder shape and contour (*k* = 0.73), as well as near perfect agreement for bladder contour (*k* = 0.82).

**Table 2:** Proposed classification system for bladder shape.

Characteristic	Options
Shape	Round
	Oblong
	Christmas tree
Contour	Smooth
	Trabeculated/diverticulated

### 3.3 Bladder Filling Pressures

**Figure 2:**
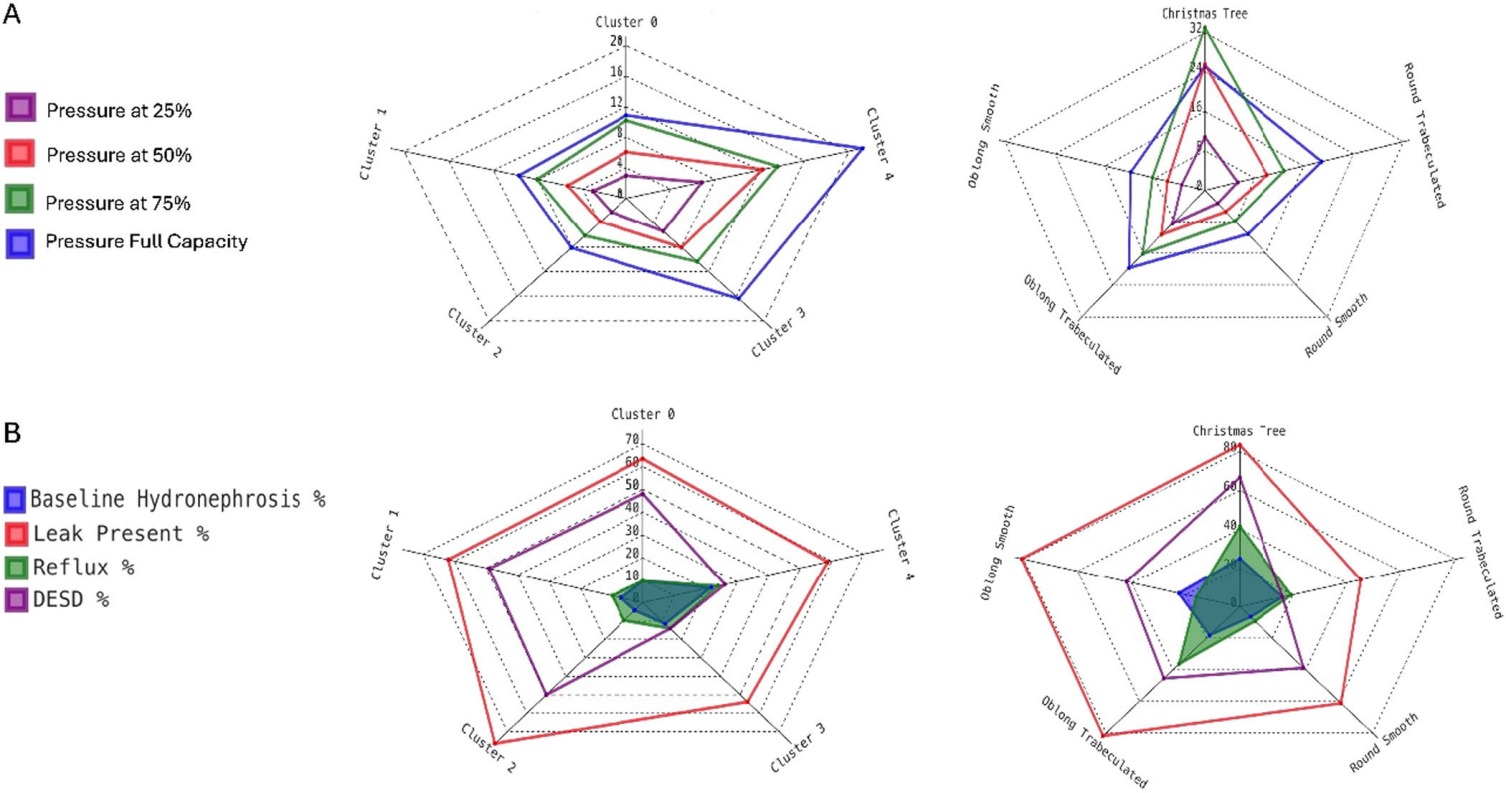
Radar plots comparing (A) mean bladder pressure at 25%, 50%, 75%, and full estimated bladder capacity between bladder shape clusters (left side) and bladder shape subjective categories (right side), and (B) the prevalence of hydronephrosis, vesicoureteral reflux, leakage, and DESD between bladder shape clusters (left side) and bladder shape subjective categories (right side). The numbers used to generate this figure along with the *p* values for pairwise statistical comparisons between different shapes are reported in Supplemental Tables S1–S8. DESD = detrusor‐external sphincter dyssynergia.

There were differences in filling pressures noted among bladder shape clusters and clinician‐determined bladder shape categories (Figure 2A). Clusters 3 and 4 (which appeared more trabeculated) had higher filling pressures than Clusters 0‐2 at most filling levels, with statistically significant differences ranging from 3 to 12 cm H2O (Supplemental Tables S1–S4). “Christmas tree”, round trabeculated, and oblong trabeculated bladders had higher filling pressures than both round and oblong smooth bladders at most filling levels, with significant differences ranging from 14 to 21 cm H2O (Supplemental Tables S1–S4). For both smooth and trabeculated bladders, there were no significant differences in filling pressures between oblong and round shapes.

### 3.4 Secondary Outcomes Reflecting Bladder Dysfunction

There were some differences in secondary outcomes reflecting bladder dysfunction among bladder shapes (Figure 2B). “Christmas tree” and oblong trabeculated bladders had VUR more frequently than round smooth bladders (41–46% vs. 10%, *p* < 0.005; Supplemental Table S5). “Christmas tree” trabeculated bladders had more hydronephrosis than round smooth bladders (29% vs. 7%, *p* < 0.005; Supplemental Table S6) and more DESD than round trabeculated bladders (67% vs. 16%, *p* < 0.005; Supplemental Table S7). Cluster 4 had VUR more frequently than Cluster 1 (24% vs. 10%, *p* = 0.037) and Cluster 2 (24% vs. 9%, *p* = 0.027). Cluster 4 had more hydronephrosis than cluster 2 (22% vs. 4%, *p* < 0.005; Supplemental Table S6), Clusters 0–2 had more DESD than Cluster 3 (49%–50% vs. 14%, *p* < 0.005; Supplemental Table S7), and Cluster 2 had more leakage than Cluster 3 (75% vs. 49%, *p* < 0.005; Supplemental Table S8). For round or oblong bladders, trabeculations did not reflect any statistically significant differences, and for smooth or trabeculated bladders, oblong shape also did not reflect any statistically significant differences.

**Figure 3:**
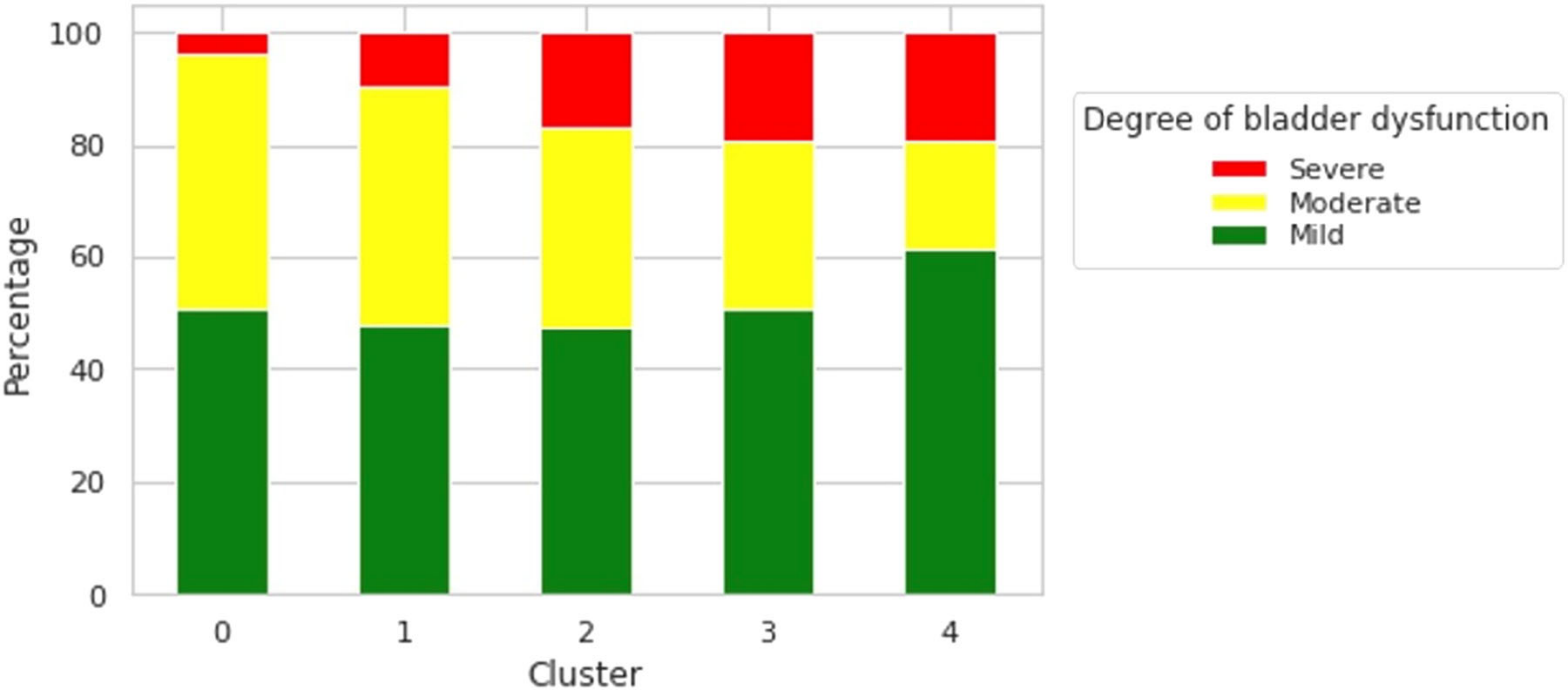
Stacked proportional bar chart showing the proportion of mild, moderate, and severe bladder dysfunction for each bladder shape cluster.

Of the 284 VUDS studies with bladder dysfunction ratings, the degree of bladder dysfunction was none to mild in 50%, moderate in 36%, and severe in 14%. There was an observed trend of worsening bladder dysfunction from clusters 0–4, although this trend was moderate, and severely dysfunctional bladders (4%–19%) were observed across all clusters (Figure 3). Cluster 4 had the highest proportions of both mild (61%) and severe (19%) bladder dysfunction compared to other clusters.

“Christmas tree” and oblong trabeculated bladders had the highest median pressures at the time of leakage during VUDS (43 and 49 cm H2O, respectively; *vs.* 35–39 cm H2O with other bladder shapes) (Supplemental Table S9). However, Clusters 3 and 4 (which appeared more trabeculated) had similar median pressures at the time of leakage (40 and 35 cm H2O respectively) compared to other clusters (35–44 cm H2O). “Christmas tree” trabeculated bladders had the lowest median % EBC at which leakage occurred (26% EBC, vs. 54%–65% EBC for other shapes). Overall, regardless of the classification method, bladder shapes had large interquartile ranges for pressure at leak (approximately 30–50 cm H2O) and % EBC at leak (approximately 20%–50%).

### 3.5 Sensitivity Analyses

When patients with a history of anticholinergic medication, CIC, bladder Botox, or ureteral reimplantation were excluded (*n* = 126 after exclusion), results of pairwise comparisons were similar to the original analysis (Supplemental Figure S1). When bladders were stratified by the overall degree of dysfunction on VUDS (none to mild, moderate, and severe), there were no significant differences in VUR, leak, DESD, and hydronephrosis between bladder shapes with the same degree of dysfunction. VUR and hydronephrosis generally increased across all clusters with increasing bladder dysfunction (Supplemental Figure S2). When using silhouette analysis with the cluster analysis, the optimal number of clusters (K) was determined to be 3, and selected secondary outcomes were observed to be comparable between the 3 clusters (Supplemental Figure S3).

## 4 Discussion

As professional societies recommend better defining the benefits of fluoroscopic exposure during urodynamic studies, there is a need to standardize bladder shape terminology to understand the diagnostic and prognostic value of bladder shape. In the present study, we propose standardized terminology for bladder shape in children and adolescents with spina bifida and explore the relationship between fluoroscopic bladder shape and clinical parameters reflecting bladder dysfunction. Our proposed nomenclature describes both bladder shape (round, oblong, or “Christmas tree”) and contour (smooth or trabeculated/diverticulated). We found that “Christmas tree” shape and trabeculated contour are associated with increased bladder filling pressures. While there are some differences in the presence of hydronephrosis, vesicoureteral reflux, leakage, detrusor‐external sphincter dyssynergia, and bladder dysfunction severity among bladder shapes, the clinical significance of these differences in unclear. Our proposed nomenclature to standardize bladder shape assessment adds to previous literature by providing specific criteria to assess both bladder shape and contour while allowing enough granularity to capture the range of bladder shapes encountered in the spina bifida population. This standardization may help facilitate further research on the role of bladder shape in predicting long‐term renal and bladder outcomes.

In both children and adults with neurogenic and non‐neurogenic voiding dysfunction, there have been some observed associations between bladder shape and clinical outcomes, although the applicability of these studies has been limited by non‐standardized bladder shape assessment. One study of children with spina bifida found that greater height‐to‐width ratio (i.e., more oblong shape) was associated with elevated end‐filling pressure and detrusor leak point pressure [11]. However, trabeculations were not considered and may have been more prevalent in more oblong bladders. Among children with neurogenic bladder, severe bladder trabeculation has been postulated to reflect intrinsic outlet resistance and therefore predict continence after bladder augmentation without an associated bladder neck procedure [12]. In adults with neurogenic bladder, trabeculations and “pine tree” shape have been associated with lower bladder compliance and development of hydronephrosis and/or VUR [2]. In men with non‐neurogenic lower urinary tract symptoms, a cone‐shaped bladder (as opposed to a round bladder) on magnetic resonance imaging was reflective of younger age, less severe urinary symptoms, and higher peak urinary flow [13]. The present study adds to our understanding of the clinical importance of bladder shape and establishes terminology that may be used to describe specific bladder shapes across multiple populations. Prior work demonstrated a strong intra‐class correlation coefficient of 0.99 for grading the degree of bladder trabeculation [3], as well as Spearman correlation coefficients of 0.72 for overall bladder shape (“smooth and round” or not) and 0.69 for the bladder neck (open or closed) [4]. Our study demonstrates that classifying both bladder shape and contour together (*k* = 0.73) can be done with substantial inter‐rater reliability.

We found that bladder shape alone does not consistently differentiate the degree of concurrent bladder dysfunction and upper tract sequelae in children with spina bifida. For example, the relatively high proportions of both mildly and severely dysfunctional bladders in Cluster 4 suggest that factors other than shape may be more influential in determining the overall degree of bladder dysfunction. It is possible that bladder pressure may be a more influential factor in this setting. In our study, the greater prevalence of VUR in “Christmas tree” and oblong trabeculated bladders may reflect their observed higher filling pressures. In the literature, detrusor leak point pressure greater than 40 cm H2O has been associated with VUR, hydronephrosis, and decreased renal function in patients with spina bifida [14, 15]. Longitudinally, detrusor pressure at initial catheterization during VUDS, end‐filling pressure, and elevated detrusor leak point pressure have all been associated with the development of renal scan abnormalities [16, 17]. Elevated detrusor leak point pressure has also been associated with the development of moderate to severe hydronephrosis, absence of renal growth, and smaller renal size [18]. Detrusor leak point pressure has also been found to better predict hydronephrosis than several other risk factors, including low compliance, high pressure detrusor contractions, DESD, and significant VUR [19]. In the present study, “Christmas tree” bladders had high detrusor leak point pressures at relatively low filling volumes, suggesting that this shape reflects poor storage ability and overall bladder function. However, notable variation in leak point pressures for any given bladder shape suggests that shape alone is not always indicative of pressure and subsequent outcomes.

Despite the absence of clear correlations between bladder shape and the presence of vesicoureteral reflux, leakage, detrusor‐external sphincter dyssynergia, and hydronephrosis in the present study, there remain several potential applications of bladder shape. Fluoroscopy during VUDS remains valuable for evaluating the bladder neck, identifying concurrent VUR and DESD, and assessing overall bladder anatomy. Second, the possibility of using fluoroscopic bladder shape on VCUG to triage pediatric spina bifida patients for VUDS has also been proposed [11, 20]. However, our finding that moderate and severe bladder dysfunction are present among most bladder shapes suggests that shape alone should not be used to triage patients for pressure‐flow studies. It remains to be determined if bladder shape may be used in combination with other clinical factors (such as bladder pressures, hydronephrosis, and leakage) to identify clinically important outcomes, such as the risk of worsening bladder dysfunction, time to deterioration, response to interventions, and recoverability of bladder function. These factors may influence decisions such as when to initiate clean intermittent catheterization (or increase its frequency) or when to proceed with bladder augmentation.

Strengths of the study including a relatively large sample size, methods using both unsupervised cluster analysis to remove clinician biases as well as clinician categorization to maintain clinical relevance, and consistent findings between both bladder shape classification approaches. While the only other study to assess bladder shape with AI used principal component analysis (dimensionality reduction) to identify modes of variation among bladder shapes (ex: left side, right side, all axes), we used cluster analysis in the present study to group similar data points and provide clusters as a more visually understandable and generalizable output. However, several limitations are worthy of mention. First, the cross‐sectional analysis has limited causal inference for the relationship between bladder shape and clinical parameters. Second, most patients in the cohort had mildly to moderately dysfunctional bladders. It is possible that inclusion of more severely dysfunctional bladders may have increased the frequency of the clinical outcomes for certain bladder shapes. Third, we could not reliably account for the durations of prior or current interventions (CIC, anticholinergics, etc.) or treatment compliance, which could potentially influence bladder shape. However, we attempted to minimize these effects by selecting images from the first available VUDS study and performing a sensitivity analysis excluding patients with prior treatment history, and only 2% of the cohort was on CIC. Fourth, bladder neck appearance was not incorporated into bladder shape assessment, as we were unable to reliably determine when an open‐appearing bladder neck on fluoroscopy was pathogenic. (e.g., an open‐appearing bladder neck may be normal if occurring simultaneously with an uninhibited bladder contraction without leakage.) Fifth, for “Christmas tree” bladders, lower filling pressures at EBC were likely due to early cessation of filling in higher‐pressure bladders, which led to selection for lower‐pressure bladders at EBC. Finally, we were unable to explain differences between clusters determined by machine learning that may correlate with differences in outcomes (“black box” effect). For example, it is possible that the size of the bladder in the fluoroscopic image may have influenced the machine learning clustering algorithm in addition to shape characteristics.

These limitations guide the future studies on bladder shape. Based on our results that round and oblong bladders generally have similar filling pressures and clinical outcomes, the distinction between round and oblong may not be necessary in bladder shape classification pending further study. Our classification methods require external validation and calibration in other populations. Future studies may use a combination of supervised learning (e.g., training an algorithm based on data labeled by experts) and unsupervised learning to improve computer‐based classification of bladder shape. Longitudinal studies may assess the effects of interventions such as anticholinergics or CIC on bladder shape over time and whether changes in shape correspond with clinical improvements. Finally, the use of standardized terminology as proposed may allow for better comparison of the significance of bladder shape across multiple studies.

## 5 Conclusion

We proposed a standardized nomenclature for bladder shape and used it to classify fluoroscopic bladder images in 417 children and adolescents with spina bifida. Certain bladder shape characteristics (trabeculations, “Christmas tree” appearance, but not oblong shape) were associated with higher filling pressures on video urodynamics. While there were some differences in upper and lower urinary tract findings reflecting bladder dysfunction among bladder shapes, these differences have uncertain clinical importance. Standardization of bladder shapes may facilitate further research on the utility of bladder shape in predicting long‐term bladder dysfunction outcomes. Regarding machine‐learning‐based interpretation of bladder shape, future research using supervised machine learning is necessary to determine the accuracy of this approach, and an improved understanding of the significance of bladder shape alongside other clinical and urodynamic data is needed to determine the feasibility of this approach for guiding clinical decision making in pediatric neuro‐urology.

## Ethics Statement

This study used deidentified data and was therefore deemed exempt from review by the Institutional Review Board of The Children's Hospital of Philadelphia.

## Consent

Consent was not necessary, as this study used de‐identified data and was deemed exempt from IRB review.

## Conflicts of Interest

Dr. Stephen A. Zderic is a cofounder of and scientific adviser to UroGenie with funding from NIH SBIR R44 DK127835. The remaining authors have no disclosures.

## Supporting information

Supplementary figures. **Supplemental Figure S1:** Sensitivity analysis excluding patients with a history of anticholinergic medication, CIC, bladder Botox, or ureteral reimplantation. **Supplemental Figure S2:** Distribution of hydronephrosis, vesicoureteral reflux, and leakage among bladder shape clusters for bladders with mild, moderate, and severe dysfunction. **Supplemental Figure S3:** Distribution of hydronephrosis, vesicoureteral reflux, and leakage stratified by bladder shape clusters with K=3.

Supplemental Tables 5‐7‐25. **Supplemental Table S1:** Comparison of mean bladder pressure at 25% EBC between bladder shapes using the Wilcoxon‐Mann‐Whitney test. **Supplemental Table S2:** Comparison of mean bladder pressure at 50% EBC between bladder shapes using the Wilcoxon‐Mann‐Whitney test. **Supplemental Table S3:** Comparison of mean bladder pressure at 75% EBC between bladder shapes using the Wilcoxon‐Mann‐Whitney test. **Supplemental Table S4:** Comparison of mean bladder pressure at EBC between bladder shapes using the Wilcoxon‐Mann‐Whitney test. **Supplemental Table S5:** Comparison of the proportion of patients with vesicoureteral reflux between bladder shapes using the Chi‐square test. **Supplemental Table S6:** Comparison of the proportion of patients with hydronephrosis between bladder shapes using the Chi‐square test. **Supplemental Table S7:** Comparison of detrusor external sphincter dyssynergia (DESD) between bladder shapes using the Chi‐square test. **Supplemental Table S8:** Comparison of the proportion of patients with leakage during video urodynamics between bladder shapes using the Chi‐square test. **Supplemental Table S9:** Filling pressure and proportion of estimated bladder capacity at which leakage occurred during video urodynamics for different bladder shapes.

Supmat.

## Data Availability

The data that support the findings of this study are available from the corresponding author upon reasonable request. The data sets generated during and/or analyzed during the current study are available from the corresponding author on reasonable request.
