# Supplementary figures and images for: Evaluation of Bladder Dysfunction Outcomes Among Standardized Bladder Shapes in Children With Spina Bifida
Source: Neurourol Urodyn. 2025 Aug 25;44(8):1560–8. doi: 10.1002/nau.70131 (PMC12550354; doi:10.1002/nau.70131)

Supplementary figure 1:


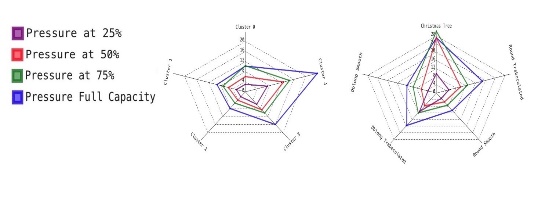


Supplementary figure 2:


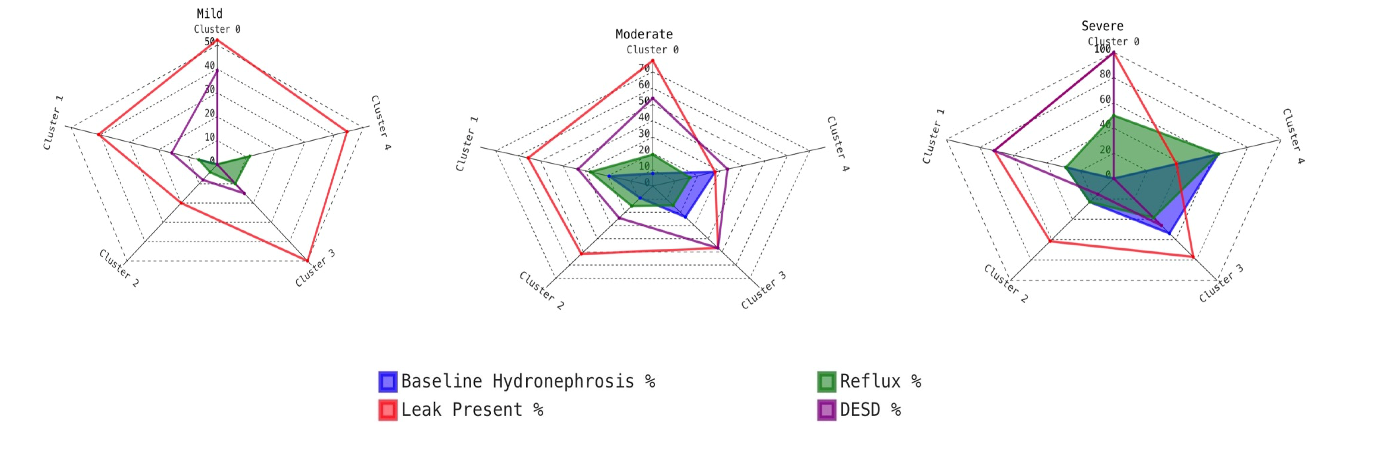


Supplementary figure 3:


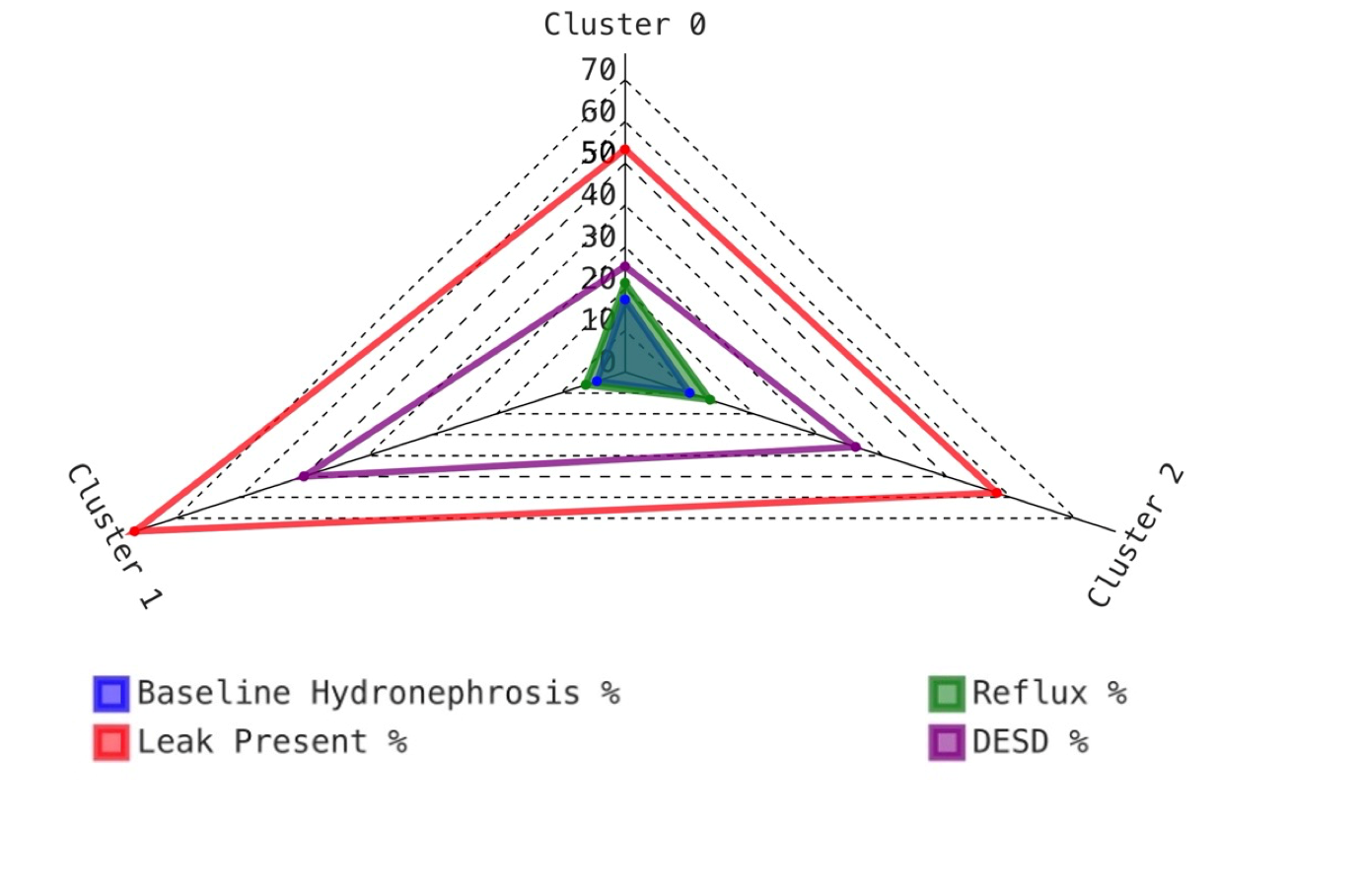

Supplement: Supplementary file 1 — Supplementary figures. Supplemental Figure S1: Sensitivity analysis excluding patients with a history of anticholinergic medication, CIC, bladder Botox, or ureteral reimplantation. Supplemental Figure S2: Distribution of hydronephrosis, vesicoureteral reflux, and leakage among bladder shape clusters for bladders with mild, moderate, and severe dysfunction. Supplemental Figure S3: Distribution of hydronephrosis, vesicoureteral reflux, and leakage stratified by bladder shape clusters with K=3. [file NAU-44-1560-s003.docx]
