# Supplementary material for: Evaluation of Bladder Dysfunction Outcomes Among Standardized Bladder Shapes in Children With Spina Bifida
Source: Neurourol Urodyn. 2025 Aug 25;44(8):1560–8. doi: 10.1002/nau.70131 (PMC12550354; doi:10.1002/nau.70131)
Supplement: Supplementary file 2 — Supplemental Tables 5‐7‐25. Supplemental Table S1: Comparison of mean bladder pressure at 25% EBC between bladder shapes using the Wilcoxon‐Mann‐Whitney test. Supplemental Table S2: Comparison of mean bladder pressure at 50% EBC between bladder shapes using the Wilcoxon‐Mann‐Whitney test. Supplemental Table S3: Comparison of mean bladder pressure at 75% EBC between bladder shapes using the Wilcoxon‐Mann‐Whitney test. Supplemental Table S4: Comparison of mean bladder pressure at EBC between bladder shapes using the Wilcoxon‐Mann‐Whitney test. Supplemental Table S5: Comparison of the proportion of patients with vesicoureteral reflux between bladder shapes using the Chi‐square test. Supplemental Table S6: Comparison of the proportion of patients with hydronephrosis between bladder shapes using the Chi‐square test. Supplemental Table S7: Comparison of detrusor external sphincter dyssynergia (DESD) between bladder shapes using the Chi‐square test. Supplemental Table S8: Comparison of the proportion of patients with leakage during video urodynamics between bladder shapes using the Chi‐square test. Supplemental Table S9: Filling pressure and proportion of estimated bladder capacity at which leakage occurred during video urodynamics for different bladder shapes. [file NAU-44-1560-s002.docx]

Supplemental Table S1. Comparison of mean bladder pressure at 25% EBC between bladder shapes using the Wilcoxon-Mann-Whitney test. P-value < 0.005 (Bonferroni-corrected P-value) was considered significant; significant P-values are indicated in bold.

| Method of bladder shape classification | Comparison Group 1 | Comparison Group 1 mean  pressure at 25% EBC (cm H2O) | Comparison Group 2 | Comparison Group 2 mean  pressure at 25% EBC (cm H2O) | P-value |
| --- | --- | --- | --- | --- | --- |
| Machine learning | Cluster 0 | 3.0 | Cluster 1 | 3.3 | 0.72 |
|  |  |  | Cluster 2 | 2.3 | 0.097 |
|  |  |  | Cluster 3 | 5.6 | **9.7 × 10⁻⁶** |
|  |  |  | Cluster 4 | 6.8 | **1.4 × 10⁻⁴** |
|  | Cluster 1 | 3.3 | Cluster 2 | 2.3 | 0.013 |
|  |  |  | Cluster 3 | 5.6 | **2.0 × 10⁻⁷** |
|  |  |  | Cluster 4 | 6.8 | **3.5 × 10⁻⁵** |
|  | Cluster 2 | 2.3 | Cluster 3 | 5.6 | **5.6 × 10⁻¹¹** |
|  |  |  | Cluster 4 | 6.8 | **3.5 × 10⁻⁸** |
|  | Cluster 3 | 5.6 | Cluster 4 | 6.8 | 0.75 |
| Expert clinician | Christmas tree Trabeculated | 9.3 | Oblong Smooth | 3.5 | 0.024 |
|  |  |  | Oblong Trabeculated | 10.2 | 0.74 |
|  |  |  | Round Smooth | 3.4 | **0.0058** |
|  |  |  | Round Trabeculated | 5.8 | 0.33 |
|  | Oblong Smooth | 3.5 | Oblong Trabeculated | 10.2 | **0.0022** |
|  |  |  | Round Smooth | 3.4 | 0.93 |
|  |  |  | Round Trabeculated | 5.8 | 0.034 |
|  | Oblong Trabeculated | 10.2 | Round Smooth | 3.4 | **1.7 × 10⁻⁴** |
|  |  |  | Round Trabeculated | 3.3 | 0.12 |
|  | Round Smooth | 3.0 | Round Trabeculated | 2.3 | **0.0026** |

Supplemental Table S2. Comparison of mean bladder pressure at 50% EBC between bladder shapes using the Wilcoxon-Mann-Whitney test. P-value < 0.005 (Bonferroni-corrected P-value) was considered significant; significant P-values are indicated in bold.

| Method of bladder shape classification | Comparison Group 1 | Comparison Group 1 mean  pressure at 50% EBC  (cm H2O) | Comparison Group 2 | Comparison Group 2 mean pressure at 50% EBC  (cm H2O) | P-value |
| --- | --- | --- | --- | --- | --- |
| Machine learning | Cluster 0 | 6.2 | Cluster 1 | 5.8 | 0.76 |
|  |  |  | Cluster 2 | 3.8 | 0.056 |
|  |  |  | Cluster 3 | 8.5 | **6.4 × 10⁻³** |
|  |  |  | Cluster 4 | 11.4 | **2.9 × 10⁻³** |
|  | Cluster 1 | 5.8 | Cluster 2 | 3.8 | 0.015 |
|  |  |  | Cluster 3 | 8.5 | **2.9 × 10⁻⁵** |
|  |  |  | Cluster 4 | 11.4 | **3.7 × 10⁻⁵** |
|  | Cluster 2 | 3.8 | Cluster 3 | 8.5 | **8.1 × 10⁻¹⁰** |
|  |  |  | Cluster 4 | 11.4 | **3.3 × 10⁻⁸** |
|  | Cluster 3 | 8.5 | Cluster 4 | 11.4 | 0.21 |
| Expert clinician | Christmas tree Trabeculated | 24.4 | Oblong Smooth | 5.8 | **2.9 × 10⁻⁴** |
|  |  |  | Oblong Trabeculated | 11.2 | 0.14 |
|  |  |  | Round Smooth | 5.5 | **9.7 × 10⁻⁶** |
|  |  |  | Round Trabeculated | 10.7 | 0.032 |
|  | Oblong Smooth | 5.8 | Oblong Trabeculated | 11.2 | **4.7 × 10⁻³** |
|  |  |  | Round Smooth | 5.8 | 0.72 |
|  |  |  | Round Trabeculated | 3.8 | **1.4 × 10⁻³** |
|  | Oblong Trabeculated | 6.2 | Round Smooth | 8.5 | **5.5 × 10⁻⁴** |
|  |  |  | Round Trabeculated | 11.4 | 0.81 |
|  | Round Smooth | 5.8 | Round Trabeculated | 3.8 | **4.2 × 10⁻⁶** |

Supplemental Table S3. Comparison of mean bladder pressure at 75% EBC between bladder shapes using the Wilcoxon-Mann-Whitney test. P-value < 0.005 (Bonferroni-corrected P-value) was considered significant; significant P-values are indicated in bold.

| Method of bladder shape classification | Comparison Group 1 | Comparison Group 1 mean  pressure at 75% EBC  (cm H2O) | Comparison Group 2 | Comparison Group 2 mean pressure at 75% EBC  (cm H2O) | P-value |
| --- | --- | --- | --- | --- | --- |
| Machine learning | Cluster 0 | 10.6 | Cluster 1 | 7.8 | 0.24 |
|  |  |  | Cluster 2 | 6 | 0.014 |
|  |  |  | Cluster 3 | 10.9 | 0.27 |
|  |  |  | Cluster 4 | 13.6 | 0.055 |
|  | Cluster 1 | 7.8 | Cluster 2 | 6 | 0.053 |
|  |  |  | Cluster 3 | 10.9 | **2.3 × 10⁻³** |
|  |  |  | Cluster 4 | 13.6 | **1.1 × 10⁻⁴** |
|  | Cluster 2 | 6 | Cluster 3 | 10.9 | **2.4 × 10⁻⁶** |
|  |  |  | Cluster 4 | 13.6 | **3.6 × 10⁻⁶** |
|  | Cluster 3 | 10.9 | Cluster 4 | 13.6 | 0.091 |
| Expert clinician | Christmas tree Trabeculated | 28.8 | Oblong Smooth | 8.4 | **5.0 × 10⁻³** |
|  |  |  | Oblong Trabeculated | 16.1 | 0.18 |
|  |  |  | Round Smooth | 7.8 | **1.6 × 10⁻³** |
|  |  |  | Round Trabeculated | 14.7 | 0.053 |
|  | Oblong Smooth | 8.4 | Oblong Trabeculated | 16.1 | **2.7 × 10⁻³** |
|  |  |  | Round Smooth | 7.8 | 0.53 |
|  |  |  | Round Trabeculated | 14.7 | 0.014 |
|  | Oblong Trabeculated | 16.1 | Round Smooth | 7.8 | **9.9 × 10⁻⁵** |
|  |  |  | Round Trabeculated | 14.7 | 0.43 |
|  | Round Smooth | 7.8 | Round Trabeculated | 14.7 | **1.3 × 10⁻⁴** |

Supplemental Table S4. Comparison of mean bladder pressure at EBC between bladder shapes using the Wilcoxon-Mann-Whitney test. P-value < 0.005 (Bonferroni-corrected P-value) was considered significant; significant P-values are indicated in bold.

| Method of bladder shape classification | Comparison Group 1 | Comparison Group 1 mean  pressure at EBC (cm H2O) | Comparison Group 2 | Comparison Group 2 mean pressure at EBC  (cm H2O) | P-value |
| --- | --- | --- | --- | --- | --- |
| Machine learning | Cluster 0 | 10.9 | Cluster 1 | 10.3 | 0.78 |
|  |  |  | Cluster 2 | 8.1 | 0.91 |
|  |  |  | Cluster 3 | 17 | **4.3 × 10⁻⁶** |
|  |  |  | Cluster 4 | 20.5 | **3.1 × 10⁻³** |
|  | Cluster 1 | 10.3 | Cluster 2 | 8.1 | 0.58 |
|  |  |  | Cluster 3 | 17 | **7.7 × 10⁻⁸** |
|  |  |  | Cluster 4 | 20.5 | **1.0 × 10⁻³** |
|  | Cluster 2 | 8.1 | Cluster 3 | 17 | **1.3 × 10⁻⁹** |
|  |  |  | Cluster 4 | 20.5 | **1.3 × 10⁻⁴** |
|  | Cluster 3 | 17 | Cluster 4 | 20.5 | 0.58 |
| Expert clinician | Christmas tree Trabeculated | 25.5 | Oblong Smooth | 11.5 | **3.8 × 10⁻³** |
|  |  |  | Oblong Trabeculated | 21.7 | 0.68 |
|  |  |  | Round Smooth | 11.3 | **3.4 × 10⁻⁴** |
|  |  |  | Round Trabeculated | 20 | 0.32 |
|  | Oblong Smooth | 11.5 | Oblong Trabeculated | 21.7 | 0.013 |
|  |  |  | Round Smooth | 11.3 | 0.76 |
|  |  |  | Round Trabeculated | 20 | **7.6 × 10⁻⁴** |
|  | Oblong Trabeculated | 21.7 | Round Smooth | 11.3 | **3.7 × 10⁻³** |
|  |  |  | Round Trabeculated | 20 | 0.65 |
|  | Round Smooth | 11.3 | Round Trabeculated | 20 | **1.5 × 10⁻⁶** |

Supplemental Table S5. Comparison of the proportion of patients with vesicoureteral reflux between bladder shapes using the Chi-square test. P-value < 0.005 (Bonferroni-corrected P-value) was considered significant; significant P-values are indicated in bold.

| Method of bladder shape classification | Comparison Group 1 | Comparison Group 1  reflux | Comparison Group 2 | Comparison Group 2 reflux | P-value |
| --- | --- | --- | --- | --- | --- |
| Machine learning | Cluster 0 | 13% | Cluster 1 | 10% | 7.47 x 10⁻¹ |
|  |  |  | Cluster 2 | 9% | 0.54 |
|  |  |  | Cluster 3 | 16% | 8.26 x 10⁻¹ |
|  |  |  | Cluster 4 | 24% | 0.22 |
|  | Cluster 1 | 10% | Cluster 2 | 9% | 0.87 |
|  |  |  | Cluster 3 | 16% | 3.19 x 10⁻¹ |
|  |  |  | Cluster 4 | 24% | **3.66 x 10⁻²** |
|  | Cluster 2 | 9% | Cluster 3 | 16% | 2.17 x 10⁻¹ |
|  |  |  | Cluster 4 | 24% | **2.73 x 10⁻²** |
|  | Cluster 3 | 16% | Cluster 4 | 24% | 0.31 |
| Expert clinician | Christmas tree Trabeculated | 41% | Oblong Smooth | 14% | 5.50 x 10⁻² |
|  |  |  | Oblong Trabeculated | 46% | 1 |
|  |  |  | Round Smooth | 10% | **4.37 x 10⁻⁴** |
|  |  |  | Round Trabeculated | 18% | 0.12 |
|  | Oblong Smooth | 14% | Oblong Trabeculated | 46% | 0.04 |
|  |  |  | Round Smooth | 10% | 7.06 x 10⁻¹ |
|  |  |  | Round Trabeculated | 18% | 8.67 x 10⁻¹ |
|  | Oblong Trabeculated | 46% | Round Smooth | 10% | **3.63 x 10⁻⁴** |
|  |  |  | Round Trabeculated | 18% | 8.63 x 10⁻² |
|  | Round Smooth | 10% | Round Trabeculated | 18% | 0.24 |

Supplemental Table S6. Comparison of the proportion of patients with hydronephrosis between bladder shapes using the Chi-square test. P-value < 0.005 (Bonferroni-corrected P-value) was considered significant; significant P-values are indicated in bold.

| Method of bladder shape classification | Comparison Group 1 | Comparison Group 1  hydronephrosis | Comparison Group 2 | Comparison Group 2 hydronephrosis | P-value |
| --- | --- | --- | --- | --- | --- |
| Machine learning | Cluster 0 | 13% | Cluster 1 | 10% | 9.78 x 10⁻¹ |
|  |  |  | Cluster 2 | 9% | 0.42 |
|  |  |  | Cluster 3 | 16% | 5.23 x 10⁻¹ |
|  |  |  | Cluster 4 | 24% | 0.071 |
|  | Cluster 1 | 10% | Cluster 2 | 9% | 0.52 |
|  |  |  | Cluster 3 | 16% | 2.11 x 10⁻¹ |
|  |  |  | Cluster 4 | 24% | **9.23 x 10⁻³** |
|  | Cluster 2 | 9% | Cluster 3 | 16% | **5.60 x 10⁻²** |
|  |  |  | Cluster 4 | 24% | **2.23 x 10⁻³** |
|  | Cluster 3 | 16% | Cluster 4 | 24% | 0.21 |
| Expert clinician | Christmas tree Trabeculated | 41% | Oblong Smooth | 14% | 6.11 x 10⁻¹ |
|  |  |  | Oblong Trabeculated | 46% | 0.64 |
|  |  |  | Round Smooth | 10% | **3.75 x 10⁻³** |
|  |  |  | Round Trabeculated | 18% | 0.37 |
|  | Oblong Smooth | 14% | Oblong Trabeculated | 46% | 1 |
|  |  |  | Round Smooth | 10% | **2.48 x 10⁻²** |
|  |  |  | Round Trabeculated | 18% | 8.77 x 10⁻¹ |
|  | Oblong Trabeculated | 46% | Round Smooth | 10% | 5.27 x 10⁻¹ |
|  |  |  | Round Trabeculated | 18% | 1 |
|  | Round Smooth | 10% | Round Trabeculated | 18% | 0.13 |

Supplemental Table S7. Comparison of detrusor external sphincter dyssynergia (DESD) between bladder shapes using the Chi-square test. P-value < 0.005 (Bonferroni-corrected P-value) was considered significant; significant P-values are indicated in bold.

| Method of bladder shape classification | Comparison Group 1 | Comparison Group 1  DESD | Comparison Group 2 | Comparison Group 2 DESD | P-value |
| --- | --- | --- | --- | --- | --- |
| Machine learning | Cluster 0 | 48% | Cluster 1 | 49% | 1 |
|  |  |  | Cluster 2 | 50% | 0.98 |
|  |  |  | Cluster 3 | 14% | **5.33 x 10⁻⁵** |
|  |  |  | Cluster 4 | 26% | 0.04 |
|  | Cluster 1 | 49% | Cluster 2 | 50% | 1 |
|  |  |  | Cluster 3 | 14% | **2.28 x 10⁻⁶** |
|  |  |  | Cluster 4 | 26% | 1.52 x 10⁻² |
|  | Cluster 2 | 50% | Cluster 3 | 14% | **5.13 x 10⁻⁶** |
|  |  |  | Cluster 4 | 26% | 1.70 x 10⁻² |
|  | Cluster 3 | 14% | Cluster 4 | 26% | 0.16 |
| Expert clinician | Christmas tree Trabeculated | 67% | Oblong Smooth | 42% | 2.65 x 10⁻¹ |
|  |  |  | Oblong Trabeculated | 45% | 0.55 |
|  |  |  | Round Smooth | 39% | 1.04 x 10⁻¹ |
|  |  |  | Round Trabeculated | 16% | **0.0042** |
|  | Oblong Smooth | 42% | Oblong Trabeculated | 45% | 1 |
|  |  |  | Round Smooth | 39% | 8.84 x 10⁻¹ |
|  |  |  | Round Trabeculated | 16% | 5.02 x 10⁻² |
|  | Oblong Trabeculated | 45% | Round Smooth | 39% | 8.97 x 10⁻¹ |
|  |  |  | Round Trabeculated | 16% | 1.21 x 10⁻¹ |
|  | Round Smooth | 39% | Round Trabeculated | 16% | 0.02 |

Supplemental Table S8. Comparison of the proportion of patients with leakage during video urodynamics between bladder shapes using the Chi-square test. P-value < 0.005 (Bonferroni-corrected P-value) was considered significant; significant P-values are indicated in bold.

| Method of bladder shape classification | Comparison Group 1 | Comparison Group 1  leakage | Comparison Group 2 | Comparison Group 2 leakage | P-value |
| --- | --- | --- | --- | --- | --- |
| Machine learning | Cluster 0 | 61% | Cluster 1 | 59% | 9.55 x 10⁻¹ |
|  |  |  | Cluster 2 | 76% | 0.08 |
|  |  |  | Cluster 3 | 49% | 2.01 x 10⁻¹ |
|  |  |  | Cluster 4 | 56% | 0.78 |
|  | Cluster 1 | 59% | Cluster 2 | 76% | 2.24 x 10⁻² |
|  |  |  | Cluster 3 | 49% | 1.80 x 10⁻¹ |
|  |  |  | Cluster 4 | 56% | 8.74 x 10⁻¹ |
|  | Cluster 2 | 76% | Cluster 3 | 49% | **4.29 x 10⁻⁴** |
|  |  |  | Cluster 4 | 56% | 2.94 x 10⁻² |
|  | Cluster 3 | 49% | Cluster 4 | 56% | 0.48 |
| Expert clinician | Christmas tree Trabeculated | 71% | Oblong Smooth | 76% | 9.51 x 10⁻¹ |
|  |  |  | Oblong Trabeculated | 77% | 1 |
|  |  |  | Round Smooth | 59% | 4.73 x 10⁻¹ |
|  |  |  | Round Trabeculated | 43% | 0.1 |
|  | Oblong Smooth | 76% | Oblong Trabeculated | 77% | 1 |
|  |  |  | Round Smooth | 59% | 6.91 x 10⁻² |
|  |  |  | Round Trabeculated | 43% | 6.53 x 10⁻³ |
|  | Oblong Trabeculated | 77% | Round Smooth | 59% | 3.07 x 10⁻¹ |
|  |  |  | Round Trabeculated | 43% | 6.61 x 10⁻² |
|  | Round Smooth | 59% | Round Trabeculated | 43% | 0.08 |

Supplemental Table S9. Filling pressure and proportion of estimated bladder capacity at which leakage occurred during video urodynamics for different bladder shapes. IQR = interquartile range.

| Method of bladder shape classification | Bladder shape | Median filling pressure (cm H2O) at which leakage occurred (IQR) | Median %EBC at which leakage occurred (IQR) |
| --- | --- | --- | --- |
| Machine learning | Cluster 0 | 35 (20-71) | 48 (37-70) |
|  | Cluster 1 | 30 (21-46) | 53 (29-77) |
|  | Cluster 2 | 44 (22-73) | 63 (37-78) |
|  | Cluster 3 | 40 (22-48) | 52 (39-89) |
|  | Cluster 4 | 35 (20-45) | 45 (26-84) |
| Expert clinician | Christmas tree Trabeculated | 43 (37-90) | 26 (23-46) |
|  | Oblong Smooth | 39 (29-52) | 54 (44-63) |
|  | Oblong Trabeculated | 49 (28-63) | 65 (45-76) |
|  | Round Smooth | 36 (20-57) | 56 (32-82) |
|  | Round Trabeculated | 35 (23-46) | 57 (52-79) |
