# Supplementary material for: Evaluation of Bladder Dysfunction Outcomes Among Standardized Bladder Shapes in Children With Spina Bifida
Source: Neurourol Urodyn. 2025 Aug 25;44(8):1560–8. doi: 10.1002/nau.70131 (PMC12550354; doi:10.1002/nau.70131)
Supplement: Supplementary file 3 — Supmat. [file NAU-44-1560-s001.docx]

Supplemental Figure S1. Sensitivity analysis excluding patients with a history of anticholinergic medication, CIC, bladder Botox, or ureteral reimplantation. Radar plots comparing mean bladder pressure at 25%, 50%, 75%, and full estimated bladder capacity between bladder shape clusters (left side) and bladder shape subjective categories (right side). DESD = detrusor-external sphincter dyssynergia.

Supplemental Figure S2. Distribution of hydronephrosis, vesicoureteral reflux, and leakage among bladder shape clusters for bladders with mild, moderate, and severe dysfunction. DESD = detrusor-external sphincter dyssynergia.

Supplemental Figure S3. Distribution of hydronephrosis, vesicoureteral reflux, and leakage stratified by bladder shape clusters with K▒=▒3. DESD = detrusor-external sphincter dyssynergia.
